# Bacterial Cooperation Causes Systematic Errors in Pathogen Risk Assessment due to the Failure of the Independent Action Hypothesis

**DOI:** 10.1371/journal.ppat.1004775

**Published:** 2015-04-24

**Authors:** Daniel M. Cornforth, Andrew Matthews, Sam P. Brown, Ben Raymond

**Affiliations:** 1 Department of Molecular Biosciences, The University of Texas, Austin, Austin, Texas, United States of America; 2 Department of Life Sciences, Imperial College London, Silwood Park, Ascot, United Kingdom; 3 Centre for Immunity, Infection and Immunity, School of Biological Sciences, University of Edinburgh, Edinburgh, United Kingdom; Stanford University, UNITED STATES

## Abstract

The Independent Action Hypothesis (IAH) states that pathogenic individuals (cells, spores, virus particles etc.) behave independently of each other, so that each has an independent probability of causing systemic infection or death. The IAH is not just of basic scientific interest; it forms the basis of our current estimates of infectious disease risk in humans. Despite the important role of the IAH in managing disease interventions for food and water-borne pathogens, experimental support for the IAH in bacterial pathogens is indirect at best. Moreover since the IAH was first proposed, cooperative behaviors have been discovered in a wide range of microorganisms, including many pathogens. A fundamental principle of cooperation is that the fitness of individuals is affected by the presence and behaviors of others, which is contrary to the assumption of independent action. In this paper, we test the IAH in *Bacillus thuringiensis (B*.*t)*, a widely occurring insect pathogen that releases toxins that benefit others in the inoculum, infecting the diamondback moth, *Plutella xylostella*. By experimentally separating *B*.*t*. spores from their toxins, we demonstrate that the IAH fails because there is an interaction between toxin and spore effects on mortality, where the toxin effect is synergistic and cannot be accommodated by independence assumptions. Finally, we show that applying recommended IAH dose-response models to high dose data leads to systematic overestimation of mortality risks at low doses, due to the presence of synergistic pathogen interactions. Our results show that cooperative secretions can easily invalidate the IAH, and that such mechanistic details should be incorporated into pathogen risk analysis.

## Author Summary

The Independent Action Hypothesis (IAH) is a basic claim in pathogen biology that underlies risk analysis for various national and international health organizations. It states that infecting pathogens act independently of one another and has proven difficult to test directly. Here we demonstrate that cooperation between infecting bacteria causes the IAH to fail in a model host-pathogen system. As a result, standard mathematical risk-assessment models, typically based on the IAH, can overestimate mortality risk at low doses. Cooperation is widespread in micro-organisms, and our results indicate that unjustified reliance on the IAH will lead to inaccurate risk assessment. Our results suggest a re-appraisal of how we assess risk from infectious agents, and for the development of mechanistic, pathogen-specific models.

## Introduction

In even the best studied host-pathogen systems, the exact relation between the inoculum size and the probability of disease is unclear. This “dose-response” relationship is not only of basic scientific interest [1,2] but is also important to accurately gauge disease risk in exposed human and livestock populations [3–10]. Unfortunately direct evaluation of disease rates at relevant pathogen doses can be unethical in humans or is experimentally intractable: at very low doses most experiments lack statistical power. To address this problem, data from high doses are extrapolated to lower ones by using predictive mathematical models [3–10]. These models are based on an important simplifying biological assumption: they accept the “independent action hypothesis.”

The independent action hypothesis has two components. It states “(a) that bacteria act independently after inoculation, and (b) a mean probability (1 > p > 0) per inoculated bacterium of initiating a fatal infection which is constant and unaffected by the number of bacteria inoculated” [11–13]. The first of these claims is suspect in light of the fast-growing list of known cooperative behaviors in bacteria, like the widespread ability of bacteria to collectively alter their shared environment by secreting toxins, exo-enzymes and iron-scavenging molecules [14]. The second claim, on which standard dose-response models are built, is still largely accepted [3–10,15,16]. It has recently been pointed out that many epidemiological models implicitly assume this independence claim, and that this assumption can influence epidemiological dynamics [17,18]. Testing of the independent action hypothesis (IAH) has typically involved indirect inference of dose-response and co-infection experiments [1,11,15,16,19]. Though this work has been generally consistent with the IAH, to our knowledge the independent action hypothesis has never been directly confirmed nor rejected in any bacterial system.

We test the IAH with *Bacillus thuringiensis* var. *kurstaki*, a widely occurring insect pathogen [20], in larvae of the diamondback moth, *Plutella xylostella*. During sporulation each bacterium produces a proteinaceous toxin crystal (Cry toxin). When a group of bacteria is ingested, these crystals are solubilised in the midgut and perforate it, facilitating host invasion and septicaemic proliferation in the haeomolymph [21]. These toxin crystals are ‘public goods’ because the toxins produced by any single cell can benefit all the cells in the midgut [22]. By independently manipulating toxin dose and bacterial density, we are able to demonstrate that toxins and spores interact to determine mortality and that toxins exhibit a threshold-like effect on mortality, thus invalidating the second component of the IAH. We then demonstrate that this failure leads to a systematic overestimation of infection risks at low doses by simulating the recommended dose-response procedures that rely on IAH-based mathematical models. This work demonstrates that bacterial cooperation can invalidate the independent action hypothesis and more generally that the formulation of risk assessment models should be driven by careful consideration of mechanisms of pathogenesis.

## Results and Discussion

We studied the contributions of *B*. *thuringiensis (B*.*t*.*)* spores and toxins to mortality in the diamondback moth *(P*. *xylostella*) by infecting larvae with inocula of varying doses of plasmid-cured mutants, lacking genes for toxin production, combined with *B*. *t*. toxins produced by recombinant *Escherichia coli* (see methods). Although the solubilized Cry toxins lead to cooperative interactions inside the larval host [22], it would in principle still be possible that each bacterium has an independent probability of killing the host if the following conditions were both met: i) if the mortality effects of toxins and spores were independent of one another and ii) if the dose response of toxins themselves fit independent action assumptions. If these were both true then each bacterium could be ascribed an independent likelihood of causing mortality via the added effects of its toxins and spore. However, we will demonstrate that there is a spore toxin interaction in our system, and that the toxin effect is too threshold-like to conform to independence assumptions.

**Fig 1:**
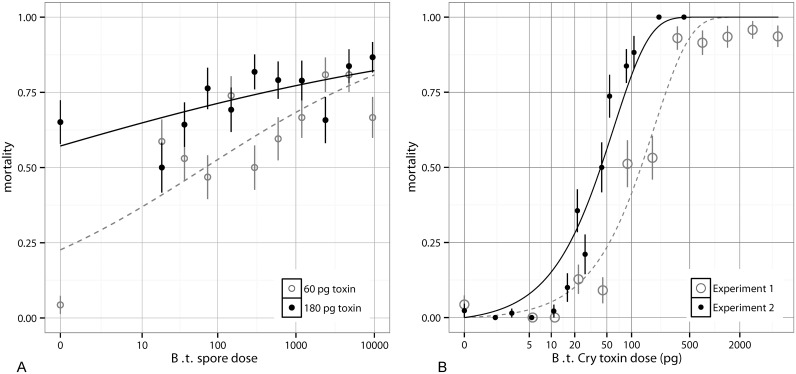
The effects of spores and toxin on mortality in *Bacillus thuringiensis*. A) The mortality rates for varying quantities of spores of *Bacillus thuringiensis* a Cry null strain supplemented with 60 pg of toxin (open circles, dashed line) or 180 pg of toxin (solid circles, solid line). A glm of the form y ~ Toxins + log(Spores+1) + Toxins* log(Spores+1) is fit and shown for both toxin doses (see S2 Table in S1 Text for parameter details) with S.E. shown. In both cases spore dose has a positive impact on mortality, and in the 60 pg case, there is a gap between zero spores and the next lowest dose. B) The mortality rates for 900 *Bacillus thuringiensis* spores supplemented with varying quantities of toxins (+/- S.E.) in two experiments. The fit curves are from a nonlinear regression with an exponential model (Eq 1). This IAH model is not sufficiently threshold-like to describe the data; it overestimates low dose mortality.

We first explored the effect of spores on mortality by fixing the toxin quantity to either 60 or 180 pg, while varying spore dose (Fig 1A). S1 Table (in S1 Text) shows a comparison of several models fit to this data; we found best support for the model y ~ Toxins + log(Spores+1)+ Toxins* log(Spores+1) based on the Akaike Information Criterion (AIC) (S1 and S2 Tables in S1 Text). Here spore dose contributed significantly to the virulence of *B*. *thuringiensis* infections (for log dose *β* = 0.37, *SE* = 0.063, *p* < 10^-8^), but this contribution was relatively minor (increasing spore dose by three orders of magnitude delivers roughly a 20% increase in mortality). There is also a negative interaction between log spores and toxins (*β* = -1.3 * 10^*–3*^, *SE* = 4.7 * 10^-4^, *p* < 10^-2^). Most evidently at 60 pg, between zero and ~14 spores there is a substantial jump in mortality rates, indicating that toxin is not solely responsible for death at low doses. This pattern of a large shift in mortality from zero to non-zero spores (at 60 pg, not 180 pg) was repeatable in subsequent toxin experiments. This is most likely because at very high toxin levels, the toxins alone are sufficient to kill the host, whereas at lower doses septicaemia is the primary cause of death and so spore quantity matters more, in line with what occurs in natural populations. At higher doses, spores contribute significantly to mortality, though as stated above, this contribution is smaller than the contribution of toxins [23].

We then conducted the reverse assay, this time fixing the spore dose at 900 and combining it with a range of doses of toxin inclusion bodies. This assay was done in two independent experiments. In this constant spore dose assay, increasing toxins greatly increased the insect mortality rate (Fig 1B). Fig 1B shows that the effect of toxins cannot be described by independent action because the data are too threshold-like. We use “threshold-like” to mean that the per-capita contribution to mortality increases with dose in the low dose range, in contrast to what independent-action models predict; we do not mean that there necessarily exists a hard threshold below which mortality cannot occur. For instance if each toxin molecule has some independent probability, *p*_0_, of killing the host, then the “exponential” dose-response model,

$$P(k)=1-e^{-p_{0}k}$$ (1)

detailed further below, describes the probability of mortality at expected dose *k*. When the data from experiment 1 are used to fit the model, the maximum likelihood fit is *p*_0_ = 0.0053. For this first dataset only points below saturation (up to 500 pg) were used to fit this model; one technical issue that could have contributed to this intermediate saturation was that very high concentrations of toxins can deter feeding, making it difficult to ensure that all insects at very high doses consumed entire droplets. The second experiment had more low-dose data points and showed no intermediate saturation; it was best fit with *p*_0_ = 0.0165. The exponential model, the steepest standard independent-action model where dose is Poisson distributed as it assumes no host variability, is unable to account for the sharp threshold-like rise (and overestimates mortality at low doses) in these experiments (Fig 1B). In S1 Fig we show that a binomial model which assumes doses are known exactly rather than being Poisson distributed, as well as the beta-Poisson which encompasses host heterogeneity (and will be described shortly), do not explain this effect either. Because the effect of toxins is too threshold-like to be described by independent action assumptions and also since the impact of toxins and spores toward mortality is non-independent, the independent action hypothesis fails in our host-pathogen system.

It is not surprising that our toxin data are inconsistent with IAH-based models at low doses since there is no *a priori* reason to expect mortality as a function of toxin to follow any particular dose-response curve without careful consideration of the mechanisms of pathogenesis. In contrast to biological dose-response curves, chemical dose-response curves often explicitly incorporate threshold-like effects [24,25]; thus when bacteria secrete toxic metabolites, the IAH can easily be violated. It is increasingly realized that the disease dynamics in *B*.*t*. and other bacteria are driven by such non-independent processes inside the host [22,26,27]. The process of pathogenesis is often implicitly discussed as either following independent action assumptions or alternatively exhibiting an absolute threshold dose below which pathogenesis or mortality never occurs [9,10]. There is an intermediate possibility, as appears to be the case in our system, where cooperative action exists but without an absolute dose cutoff below which pathogenesis is impossible.

One important application of experimental dose-response data is in determining disease risk in exposed host populations. In these exposed communities each host typically has a low likelihood of developing disease, and so the doses most relevant for public health applications are often very low. Unfortunately at these low disease rates, the data are limited and noisy so direct analysis lacks statistical power. As a result, standard practice is to determine risk at the relevant doses by extrapolating from higher dose results by using mathematical models [3–10,15,28,29]. These models are based on the assumption that each infecting cell has a constant and independent probability of causing disease. There are two commonly used models for dose response extrapolation, both based on the independent action hypothesis [6]. The “exponential model” in Eq 1 assumes each infected bacterium has a fixed probability, *p*_0_, of causing host illness or death and that the mean ingested dose is *k*. This model can be further extended to account for variation in host susceptibility with

$$P(k)=1{-}_{1}F_{1}(\alpha ,\alpha +\beta ,-k)\approx 1-{\left(1+\frac{k}{\beta }\right)}^{-\alpha }$$ (2)

where *α* and *β* are parameters for the Beta distribution describing the likelihood of infection of a host per bacterium. The exact form, which uses a confluent hypergeometric function, is nearly always approximated to the above stated “beta-Poisson model” [6], most accurate when *β > 1 and α* ≪ *β*. Generally, beta-Poisson curves reach full mortality more gradually than exponential curves because a small proportion of hosts resist extreme doses. All these IAH-based models are approximately linear at low doses and can easily overestimate risks if there are threshold-like effects, potentially driven by cooperation among infecting bacteria, as shown in Fig 1B.

**Fig 2:**
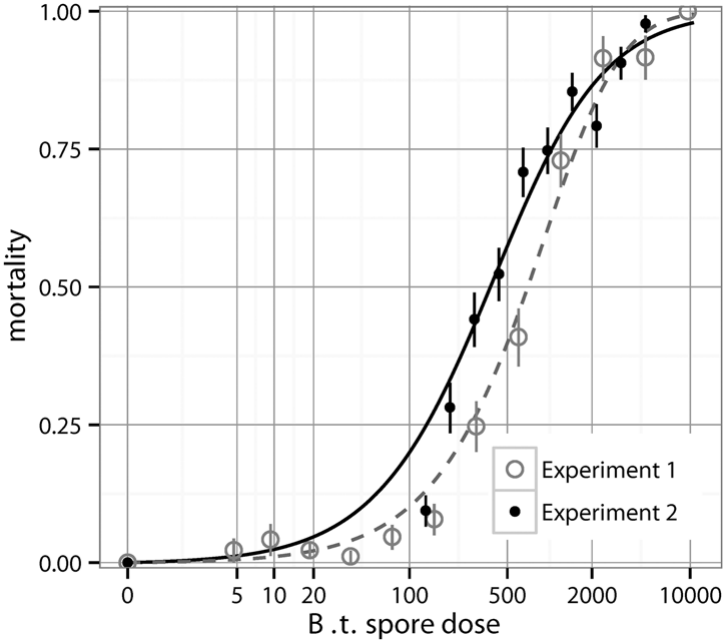
The dose-response for wild type *Bacillus thuringiensis* (+/- S.E.) in two experiments. The best IAH model (beta-Poisson) fit from all doses over 20% mortality is also shown. Again dashed line corresponds to the first experiment (with open circles), and solid line corresponds to the second experiment (with solid circles).

**Fig 3:**
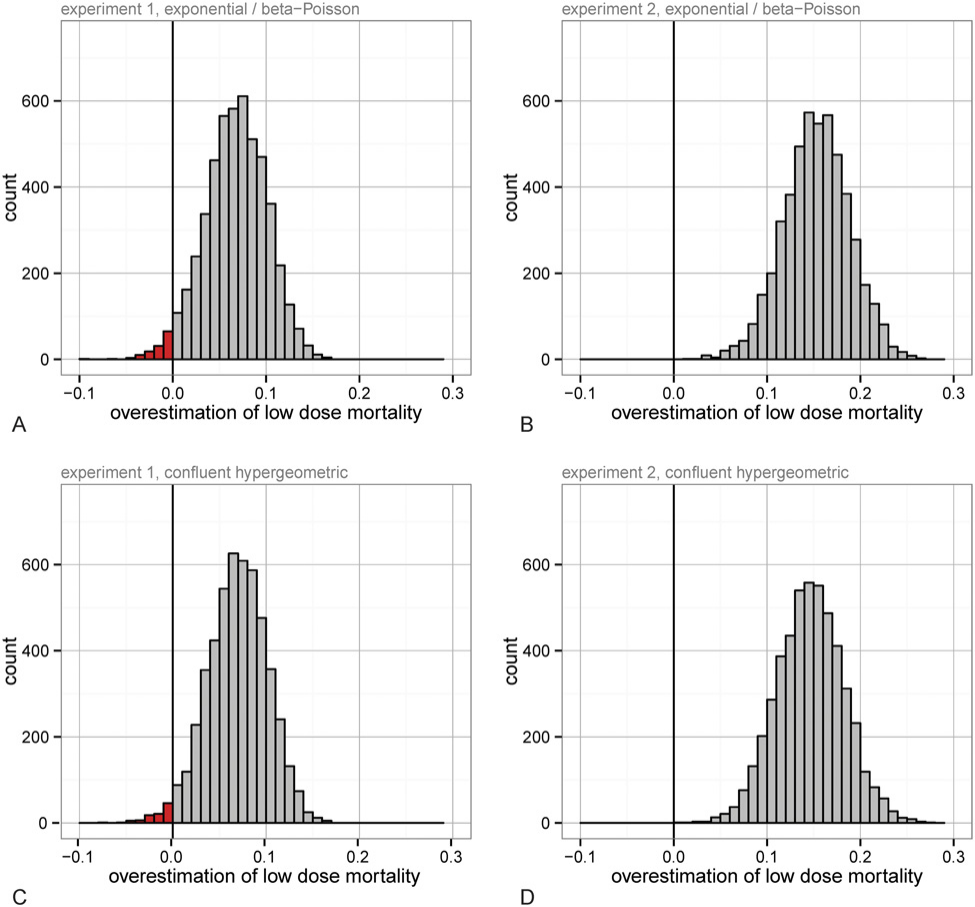
The distribution of the differences between predicted mortality and ‘actual’ mortality from best-fit models (fit to all doses over 20% mortality) at low doses in simulated experiments (generated from bootstrapping data from each dose 5,000 times). The difference between predicted and “actual” low-dose risk when the model is chosen between the best fit exponential and beta-Poisson models based on AIC for experiment 1 focusing on the spore dose of mean 150 (A) and experiment 2 focusing on the spore dose of mean 130.59 (B). The difference between predicted and “actual” low-dose risk when the less commonly used, exact (confluent hypergeometric) form is shown for data in experiment 1 (C) and experiment 2 (D).

Dose-response data and fitted models for wild-type *B*.*t* are shown in Fig 2. These data derive from two additional fully independent experiments with a total of N = 2073 larvae; see Methods for details. We used the standard methodology to determine low dose risks by assuming that we only had higher dose data for both datasets. To do this we first determined the maximum likelihood fits for the two most common dose-response curves (exponential and beta-Poisson), fitting them to all points with infection rate above 20% (all but the lowest 7 doses in experiment 1, and all but the lowest 2 doses in experiment 2). The beta-Poisson fit (shown in Fig 2) is a better fit than the exponential model as it has a lower AIC (Akaike Information Criterion). The best fit for the exponential model(*p*_0_ = 0.00089; *AIC* = 33.19 for experiment 1, *p*_0_ = 0.0012; *AIC* = 112.24 for experiment 2) is worse than the beta-Poisson model (*α* = 3.80, *β* = 3488.50; *AIC* = 30.60 for experiment 1, *α* = 1.27, *β* = 520.07; *AIC* = 56.85 for experiment 2); the beta-Poisson fits for both datasets is shown in Fig 2. In practice, a researcher would choose a model based on the data available (in our case, they would choose the beta-Poisson distribution over the exponential, based on the lower AIC at the higher doses), and then estimate risks at low doses given this best fit [3–10,15,28,30]. With the beta-Poisson model the highest dose below the points used for risk estimation is overestimated by 86.8% in experiment 1 and 160.5% in experiment 2. To more directly test the error from using IAH-based models to fit to our data we simulated the standard process of estimating the infection risk at low doses [3–10,15,28,30]. Because of the large sample sizes that would be needed for resolution at low doses, researchers are forced to use mathematical models to approximate low dose risks (in our data, infection probability less than 20%) by fitting them to the available higher doses; the particular cutoff we used did not qualitatively affect the outcome of this approach. We resampled (with replacement) all the binomial data for each dose, to produce additional simulated experiments. The process was as follows: 1) generate “pseudo-data” by resampling the actual data for each dose with replacement, 2) determine the best fit exponential and beta-Poisson model based on the high doses (above 20% infection), then pick whichever had a lower AIC, 3) use this model to extrapolate the mortality rate to the low doses, and 4) subtract the pseudo-data mortality rate at the dose of interest from the extrapolated mortality rate. When this difference is positive it indicates that the model fit overestimated risk and when negative that it underestimated the risk. We conducted this process separately in the two datasets, each with 5,000 simulated experiments. In experiment 1, the low dose predictions that differed most systematically (either underestimating or overestimating the correct risks) were at doses of 35, 75, and 150 spores where mortality was overestimated 97.8%, 88.7%, and 97.7% of the time, respectively. Although some of the other low doses were actually underestimated, none were significantly so; among the other three nonzero doses in ascending order, risk was underestimated in 64.1%, 86.2%, and 55.8% of the simulations (50% corresponds to no bias in either direction). The distribution of the differences between the extrapolated risk and true value in the resulting runs from the 150 spore dose is shown in Fig 3A. The overestimation effect in experiment 2 was more dramatic; here there was a single non-zero data point with infection rate less than 20%, with expected dose of 130.59 (from data in Fig 2); mortality was overestimated at this dose in 100% of all resampled datasets (Fig 3B). It has been noted previously that the commonly used beta-Poisson model can differ from the exact, more complicated confluent hypergeometric form from which it is derived [31]. To determine whether the same bias exists when the confluent hypergeometric model is used, we performed the same procedure using this less commonly used, non-approximated version of the model. With this exact form of the model, risk estimates were similar to in the approximated one; the risk of fatal infection at the same low doses is over-estimated 98.0% of the time in the experiment 1 data at 150 spores and in 100% of runs with the experiment 2 data at 130.59 spores (Fig 3C and 3D).

Previous approaches to test the IAH have analyzed the rate of single strain infections among hosts co-infected with differentially tagged strains at low doses. In these experiments a prevalence of clonal infections is taken as evidence that only the progeny from one inoculated cell is recovered from the final systemic infection and therefore as support for the IAH [12,16,19]. Conversely if there is not a low dose range for which clonal infection occurs, this can be evidence for a cooperative infection as in previous work with a bacterial plant pathogen infecting a non-natural host [32]. A problem with tagging methods is that they can falsely identify bottlenecks during infection as evidence for independent action [33]. Indeed bottlenecks can occur when infection success is based on cooperative interactions. More convincing is a recent extension in a virus-insect system that formalized a simple ‘null model’ for probability of mortal infection and then varied doses of each of two tagged lineages in order to test for statistical deviations from this model [1]. Among six virus-insect systems tested, two were consistent with IAH predictions, and four were not; but as their approach was statistical rather than mechanistic, the causes of these departures from IAH predictions are unknown. Some of this may be explained by recent work that has shown that heterogeneity in host susceptibilities can cause both a shallow dose-response as well as an inflated rate of mixed infection among tagged strains [34].

The application of any mechanistic dose-response model to data implicitly asserts biological claims about the system. For instance the IAH model that best fit our wild type data was the beta-Poisson model (Fig 2), which explains a relatively gradual rise in mortality with dose as being caused by heterogeneity among hosts (in contrast to the exponential model which assumes no such host heterogeneity). However if the marginal effect of additional toxins naturally diminishes as doses increase, then this leads to a slowly diminishing dose-response shape without requiring substantial host heterogeneity. So if in a model system the toxin dose-response were very shallow, applying the beta-Poisson model to the data would be implicitly claiming that there was extreme host heterogeneity even if the effect were just a property of the collective action of the toxin. One should be careful in directly comparing the insect mortalities with fixed spores and supplemented toxins (Fig 1B) to the mortality with wild type spores (Fig 2). There are differences between the toxin inclusion bodies of the wild type spores and the GM inclusion bodies produced by *E*. *coli*. Wild type inclusion bodies contain Cry1Ac, Cry1Ab, Cry1Aa and small quantities of Cry 2Aa [35] and are packaged in bipyridimal crystals, whereas the transgenic *E*. *coli* produces only the Bt toxin, Cry1Ac, which is packaged differently from the WT. Another difference was that in the fixed spores experiment (Fig 1B) the toxin was potentiated with a high and constant dose of spores (900) in each droplet, and so once there was adequate midgut perforation septicemia was nearly guaranteed. Though there are differences between the wild type and supplemented toxin dose responses, the effect of toxins is fundamentally non-independent.

We have demonstrated that the IAH fails in *B*. *thuringiensis* due to the cooperative nature of its toxins, but how common might this be in other pathogens? Closely related bacteria such as *Bacillus cereus* and *Bacillus anthracis* release a large number of diverse virulence factors [36], as do other serious human pathogenic bacteria. For instance, anthrax toxins, cholera toxin, *Staphylococcus* alpha toxin, and *Streptococcus pneumoniae* toxin are all freely released and benefit neighboring related bacteria and therefore should be expected to violate IAH assumptions [37]. It has been previously argued that there might be mechanism-based rules governing broad trends in median dose-responses [26,27,37]. Here we have extended the appeal for a mechanistic focus in dose-response from median infection risk to understanding the shapes of these dose-response curves. Though we have concentrated our efforts on shared toxins, there are other social interactions that likely have major effects on dose-responses. For instance many bacteria release extracellular enzymes and small molecules that perform many other functions including immune cell evasion, cell-to-cell signaling (i.e. quorum sensing), and biofilm formation. If a bacterium’s probability of passing a host barrier or harming the host increases with the secretions of other infecting cells [38], the main assumptions of the IAH fails and dose-response curves are likely to be affected.

We have shown that cooperative interactions between infecting pathogens can cause an error in risk assessment. What then can be done to better assess infection risk in such cases? One approach may be to construct a more detailed mechanistic model of the infection process for a pathogen of interest using dose-response data available. However this could be very difficult in practice because a cooperative effect may occur below the range of available data, making it difficult or impossible to parameterize. When the system is known to use quorum sensing, to freely release a toxin, or to exhibit another cooperative behavior, a first-level approximation may be to view the independent action calculation of infection risk as an upper ceiling. However, this should only be done with great caution because it is possible that a cooperative trait may inflate risk above that predicted by an independent action model; this could occur if the marginal effect of each released molecule showed diminishing returns across all biological concentrations rather than a threshold-like effect as we see here. In such a case with a high initial increase in mortality followed by saturation, some low doses could in principle cause a higher risk than predicted by an IAH model. A better alternative may be to utilize engineered knockout mutants of cooperative genes in order to test the impact of the specific bacterial processes on infection. Then by studying the effect of supplemented cooperative secretions, more predictive, system-specific models may be constructed.

In conclusion, our data show that cooperation of *B*. *thuringiensis* during infection of the diamondback moth *Plutella xylostella* causes the failure of the independent action hypothesis, and as a result, commonly used models overestimate disease probability at low doses. Because cooperation is a common feature of many bacteria, it is likely this overestimation extends to important human pathogens, potentially causing a misallocation of public health resources. The same biological assumptions and models have been recommended for assessing risk in bioterrorism attacks [9,39], and a criticism of the response to the 2001 anthrax attacks in the United States was that risk was grossly overestimated, costing millions in unnecessary sterilization, due to not incorporating threshold-like effects into the dose-response models [40]. Besides its implications for risk assessment, a lack of linearity in infection among low doses can significantly alter standard epidemiological assumptions for disease transmission [41] and also our understanding of genetic drift [2] and the evolution of cooperation [42] among pathogens. The articulation of the independent action hypothesis more than fifty years ago has been helpful to clarify thoughts on infection biology and risk. But the biology that has been uncovered in subsequent years questions its generality. The commonly used dose response models are used because they are simple, but given the extent of social interactions now known to occur between bacteria, the independent action hypothesis and models based on it are no longer tenable in many bacterial pathogens.

## Materials and Methods

Spontaneous antibiotic resistance mutants of *B*. *thuringienis kurstaki* HD-1, were isolated from the commercial biopesticide preparation, DiPel WP (Valent Biosciences), by plating high densities of cells (10^8^ +) on 15 *μ*g ml^-1^ nalidixic acid. An antibiotic resistant mutant with reduced fitness cost (6G Nal^R^) was isolated after a round of host passage in *P*. *xylostella* [43], and identified by rapid growth on selective plates. This strain was cured of its Cry toxin producing plasmid by growth at high temperature (42°C) and isolating colonies with unusual morphology at sporulation and in order to produce the isolate Cry null 6.20 Nal^R^. Absence of Cry toxin production was confirmed by microscopy and bioassays with *P*. *xylostella*, which confirmed that this mutant was not infectious at very high doses (>10^5^ cfu). Sporulated cultures of all strains were produced by growing dense lawns of bacteria on HCO sporulation media [44] at 30°C for 1 week. Spores and Cry toxins were recovered from plates and washed twice in sterile saline (0.85% NaCl), before being diluted into 10ml of saline and stored at -20°C in 0.5 ml aliquots for up to 8 weeks. Defrosted spores were enumerated by plating serial dilutions; replicated counts were made within 48 hours of infecting insects. Exogenous *B*. *thuringiensis* Cry toxin (Cry1Ac) was produced in *E*. *coli* JM109 cells carrying the plasmid pGem1Ac, a gift of Dr Neil Crickmore (University of Sussex). Cells were grown in 500ml of double strength LB for 3 days at 37°C with 100 *μ*g ml^-1^ ampicillin. After centrifugation (6000 g) pellets were suspended in 30 ml sterile de-ionized water and sonicated in 15ml aliquots using a Branson sonicator at 25% amplitude with four bursts of 40s with 40s rests on ice between each burst. Cells were centrifuged at 5000 g before being resuspended in water with 0.5% Triton X-100 before an additional minute of sonication. Cells were then centrifuged, and resuspended one more time before stored at -20°C in 1ml aliquots. Total Cry toxin production was estimated using SDS-PAGE and densitometry with BSA as standard using the Biorad Image Lab 4.01 software. Cry1Ac forms a strong band of 130kDa, facilitating ready quantification. There was approximately 0.6 pg of toxin per exogenous inclusion body. Toxin aliquots were pasteurized (heat treated at 65°C for 20 minutes) before use in bioassays in order to kill any remaining *E*. *coli* cells.

An inbred population of *P*. *xylostella* larvae (Geneva 88) were reared on artificial diet as described previously [45], this population has been in continuous culture for at least 20 years [46]. The parents of larvae used in bioassays were reared on artificial diet containing streptomycin and chlortetracycline. Eggs produced by these individuals were surface sterlilized with sodium hypochlorite prior to use, as described previously [45], these methods ensure that insects are largely free of enteric bacteria [45]. Larvae for assays were reared from eggs laid in standard cohorts (i.e. from the peak oviposition period 2–3 days post-mating) on antibiotic-free diet. All insects emerged from eggs onto diet within a 24 hour window. Early third instars (4–5 days old) were infected with Bt in droplet assays. We further limited size/environmental variation by only using larvae 4–5 mm in length and by excluding late second instars. Instars that are about to moult can be recognized by the dark band (the new head capsule) immediately behind the head. The final droplet mix contained 10mM sucrose, 7.5% v/v green food dye (Dr Oetker, www.oetker.co.uk) and 0.4% w/v agar (Oxoid Bacteriological), and 40% v/v cabbage extract (filtered liquid from boiled cabbages). The cabbage juice, sucrose and food dye were filter sterilized before being used to dilute the spores; this mixture was then combined (50:50) with molten 0.8% agar (at 60°C). The resultant inoculum was briefly held at 50°C in heat block while 1*μ*l droplets were dispensed into each well of 48 well plates using pre-warmed pipette tips. A single larva was added to each well, and plates were tightly sealed with damp tissue paper: larvae were allowed to feed for up to 18 hours. After feeding, larvae that had consumed at least 75% of their droplets, and which had visible green dye throughout their intestinal tract, were transferred to artificial diet for 5 days. Successful infections were classed as larvae that died and produced the strongly melanized cadavers indicative of *Bt* infection.

We carried out three sets of droplet bioassays in order to test the IAH. The first set of assays measured the response of mortality to variation in spore dose (using Cry null 6.20 Nal^R^) while holding the dose of exogenous Cry1Ac constant. This experiment was carried out at two doses of exogenous toxin (60 or 180 pg Cry1Ac) and was set up with 48 larvae per dose. The second group of assays aimed to explore the effect of increasing concentrations of the public good virulence factor (exogenous Cry1Ac toxin) while holding the spore dose constant (using Cry null 6.20 Nal^R^). These experiments used a constant spore dose of 900 CFU, while toxin dose varied from 5 pg to 6 ng; this assay was repeated and set up with 48 larvae per dose in each replicate. The final set aimed to accurately establish the shape of the dose response curve to wild-type *B*. *t*. *kurstaki* HD-1 spores and toxins using 6G Nal^R^, and we carried out two independent experiments with these wild-type spores. The first experiment used 12 doses based on a two-fold dilution series with an additional saline control, with 45–90 insects per dose. It was carried out in two blocks that were pooled into a single data set without loss of explanatory power (*F*_1,28_ = 0.5, *P* = 0.48). The second wild-type experiment was carried out to give finer resolution over a slightly higher dose range (4800–130 spores) and used ten doses diluted in a 2:1 series with 90–110 insects per dose.

Statistical analysis was performed in R v3.0.2. GLMs were calculated using the package glm2 [47]. Figures were produced using ggplot2 [48]. All nonlinear fits were using the R package bbmle (function mle2), excluding the zero dose points in the fixed spore dose-responses since the models examined assign zero likelihood at dose of zero [49]. Data deposited in the Dryad repository: http://dx.doi.org/10.5061/dryad.72f4s [50].

## Supporting Information

S1 Text Best fit parameters for the data in Fig 1A. S1 Table shows comparison of different glm fits to the data in Fig 1A. S2 Table shows the best fit parameters for the best model (based on AIC) among models in S1 Table.
(DOCX)

S1 Fig Additional independent action models fit to the toxin data shown in Fig 2B. The mortality rates for 900 *Bacillus thuringiensis* spores supplemented with varying quantities of toxins (+/- S.E.). A) The fit curve from a nonlinear regression with a binomial model (*P*(*k*) = 1- (1—*p*_0_)^*k*^), using data from both experiments (*p*_0_ = 0.00529 in experiment 1 and *p*_0_ = 0.0163 in experiment 2). The binomial dose-response model assumes no host variability, and unlike the exponential model, doses are assumed exact rather than Poisson distributed. B) The beta-Poisson approximation shown in Eq 2 (*α* = 8.65, *β* = 1549.97 in experiment 1 and *α* = 21.36, *β* = 1245.22 in experiment 2). C) The exact, confluent hypergeometric form also from Eq 2 *α* = 8.69, *β* = 1549.20 in experiment 1 and *α* = 21.39, *β* = 1244.07 for experiment 2). In the latter two models, the two parameters are highly correlated when fitting this data, yielding various parameter pairs with nearly identical curves; the fits above are consistent with MCMC runs using log-normal priors.
(TIFF)

## References

[1] Zwart MP, Hemerik L, Cory JS, de Visser JAG, Bianchi FJ, et al. (2009) An experimental test of the independent action hypothesis in virus—insect pathosystems. Proceedings of the Royal Society B: Biological Sciences: rspb. 2009.0064. 10.1098/rspb.2009.0064 PMC2677602 19324752

[2] Zwart MP, Daròs J-A, Elena SF (2011) One is enough: in vivo effective population size is dose-dependent for a plant RNA virus. PLoS pathogens 7: e1002122 10.1371/journal.ppat.1002122 21750676 PMC3131263

[3] Fazil AM (2005) A primer on risk assessment modelling: focus on seafood products: Food & Agriculture Org.

[4] Cox LA (2006) Dose-Response Modeling and Risk Characterization Quantitative Health Risk Analysis Methods: Modeling the Human Health Impacts of Antibiotics Used in Food Animals: 169–223.

[5] Buchanan† RL, Havelaar AH, Smith MA, Whiting RC, Julien* E (2009) The key events dose-response framework: its potential for application to foodborne pathogenic microorganisms. Critical reviews in food science and nutrition 49: 718–728. 10.1080/10408390903116764 19690997 PMC2840876

[6] Haas CN, Rose JB, Gerba CP (2014) Quantitative microbial risk assessment: John Wiley & Sons.

[7] Artiola J, Pepper IL, Brusseau ML (2004) Environmental monitoring and characterization: Academic Press.

[8] World Health Organization (2003) Hazard characterization for pathogens in food and water: guidelines: Food & Agriculture Org.

[9] National Research Council (2005) Reopening Public Facilities After a Biological Attack: A Decision Making Framework: National Academies Press.

[10] World Health Organization (2006) Guidelines for Drinking-water Quality, FIRST ADDENDUM TO THIRD EDITION. 28759192

[11] Meynell G (1957) The applicability of the hypothesis of independent action to fatal infections in mice given Salmonella typhimurium by mouth. Journal of general microbiology 16: 396–404. 13416517 10.1099/00221287-16-2-396

[12] Meynell G, Stocker B (1957) Some hypotheses on the aetiology of fatal infections in partially resistant hosts and their application to mice challenged with Salmonella paratyphi-B or Salmonella typhimurium by intraperitoneal injection. Journal of general microbiology 16: 38–58. 13406218 10.1099/00221287-16-1-38

[13] Schmidt PJ, Pintar KD, Fazil AM, Topp E (2013) Harnessing the Theoretical Foundations of the Exponential and Beta-Poisson Dose-Response Models to Quantify Parameter Uncertainty Using Markov Chain Monte Carlo. Risk Analysis 33: 1677–1693. 10.1111/risa.12006 23311599

[14] West SA, Griffin AS, Gardner A, Diggle SP (2006) Social evolution theory for microorganisms. Nature Reviews Microbiology 4: 597–607. 16845430 10.1038/nrmicro1461

[15] Druett H (1952) Bacterial invasion. Nature. 10.1038/170288a0 12993144

[16] Rubin LG (1987) Bacterial colonization and infection resulting from multiplication of a single organism. Review of Infectious Diseases 9: 488–493. 3299635 10.1093/clinids/9.3.488

[17] Regoes RR, Hottinger JW, Sygnarski L, Ebert D (2003) The infection rate of Daphnia magna by Pasteuria ramosa conforms with the mass-action principle. Epidemiology and Infection 131: 957–966. 14596538 10.1017/s0950268803008793 PMC2870041

[18] Ben-Ami F, Regoes RR, Ebert D (2008) A quantitative test of the relationship between parasite dose and infection probability across different host—parasite combinations. Proceedings of the Royal Society B: Biological Sciences 275: 853–859. 10.1098/rspb.2007.1544 18198145 PMC2596906

[19] Moxon ER, Murphy PA (1978) Haemophilus influenzae bacteremia and meningitis resulting from survival of a single organism. Proceedings of the National Academy of Sciences 75: 1534–1536. 306628 10.1073/pnas.75.3.1534 PMC411507

[20] Raymond B, Johnston PR, Nielsen-LeRoux C, Lereclus D, Crickmore N (2010) *Bacillus thuringiensis*: an impotent pathogen? Trends in microbiology 18: 189–194. 10.1016/j.tim.2010.02.006 20338765

[21] Schnepf E, Crickmore N, Van Rie J, Lereclus D, Baum J, et al (1998) Bacillus thuringiensis and its pesticidal crystal proteins. Microbiology and molecular biology reviews 62: 775–806. 9729609 10.1128/mmbr.62.3.775-806.1998 PMC98934

[22] Raymond B, West SA, Griffin AS, Bonsall MB (2012) The dynamics of cooperative bacterial virulence in the field. Science 337: 85–88. 10.1126/science.1218196 22767928

[23] Li R, Jarrett P, Burges H (1987) Importance of spores, crystals, and δ-endotoxins in the pathogenicity of different varieties of *Bacillus thuringiensis* in *Galleria mellonella* and *Pieris brassicae* . Journal of Invertebrate Pathology 50: 277–284.

[24] Slob W (1999) Thresholds in toxicology and risk assessment. International Journal of Toxicology 18: 259–268.

[25] World Health Organization Inter-Organization Programme for the Sound Management of Chemicals (2009) Principles for modelling dose-response for the risk assessment of chemicals: World Health Organization.

[26] Leggett HC, Cornwallis CK, West SA (2012) Mechanisms of pathogenesis, infective dose and virulence in human parasites. PLoS pathogens 8: e1002512 10.1371/journal.ppat.1002512 22359500 PMC3280976

[27] Gama JA, Abby SS, Vieira-Silva S, Dionisio F, Rocha EP (2012) Immune subversion and quorum-sensing shape the variation in infectious dose among bacterial pathogens. PLoS pathogens 8: e1002503 10.1371/journal.ppat.1002503 22319444 PMC3271079

[28] Joint FAO/WHO Expert Consultation on Risk Assessment of Microbiological Hazards in Foods: FAO headquarters, Rome, 17–21 7 2000 Rome: FAO iv, 47 p. p.

[29] Food and Agriculture Organization of the United Nations, World Health Organization (2011) Report of the Joint FAO/WHO Expert Consultation on the Risks and Benefits of Fish Consumption: Rome, 25–29 1 2010 Rome: Food and Agriculture Organization of the United Nations: World Health Organization x, 50 p. p.

[30] Rose JB, Haas CN, Gurian PL, Koopman JS (2008) Instruction Manual for Quantitative Microbial Risk Assessment (QMRA).

[31] Teunis P, Havelaar A (2000) The Beta Poisson Dose-Response Model Is Not a Single-Hit Model. Risk Analysis 20: 513–520. 11051074 10.1111/0272-4332.204048

[32] Ercolani G (1973) Two hypotheses on the aetiology of response of plants to phytopathogenic bacteria. Journal of General Microbiology 75: 83–95.

[33] Grant AJ, Restif O, McKinley TJ, Sheppard M, Maskell DJ, et al (2008) Modelling within-host spatiotemporal dynamics of invasive bacterial disease. PLoS biology 6: e74 10.1371/journal.pbio.0060074 18399718 PMC2288627

[34] van der Werf W, Hemerik L, Vlak JM, Zwart MP (2011) Heterogeneous host susceptibility enhances prevalence of mixed-genotype micro-parasite infections. PLoS computational biology 7: e1002097 10.1371/journal.pcbi.1002097 21738463 PMC3127814

[35] Höfte H, Whiteley H (1989) Insecticidal crystal proteins of Bacillus thuringiensis. Microbiological reviews 53: 242–255. 2666844 10.1128/mr.53.2.242-255.1989 PMC372730

[36] Raymond B, Bonsall MB (2013) Cooperation and the evolutionary ecology of bacterial virulence: The Bacillus cereus group as a novel study system. BioEssays 35: 706–716. 10.1002/bies.201300028 23702950

[37] Schmid-Hempel P, Frank SA (2007) Pathogenesis, virulence, and infective dose. PLoS Pathogens 3: e147. 10.1371/journal.ppat.0030147 PMC2042013 17967057

[38] Zhou L, Slamti L, Nielsen-LeRoux C, Lereclus D, Raymond B (2014) The Social Biology of Quorum Sensing in a Naturalistic Host Pathogen System. Current Biology. 10.1016/j.cub.2014.08.049 25308072

[39] Canter DA (2005) Addressing residual risk issues at anthrax cleanups: how clean is safe? Journal of Toxicology and Environmental Health, Part A 68: 1017–1032. 16020189 10.1080/15287390590912621

[40] Coleman ME, Thran B, Morse SS, Hugh-Jones M, Massulik S (2008) Inhalation anthrax: Dose response and risk analysis. Biosecurity and bioterrorism: biodefense strategy, practice, and science 6: 147–160. 10.1089/bsp.2007.0066 PMC2996252 18582166

[41] Regoes RR, Ebert D, Bonhoeffer S (2002) Dose—dependent infection rates of parasites produce the Allee effect in epidemiology. Proceedings of the Royal Society of London Series B: Biological Sciences 269: 271–279. 11839196 10.1098/rspb.2001.1816 PMC1690885

[42] Cornforth DM, Sumpter DJ, Brown SP, Brännström Å (2012) Synergy and group size in microbial cooperation. The American naturalist 180: 296 10.1086/667193 22854073 PMC3635123

[43] Garbutt J, Bonsall MB, Wright DJ, Raymond B (2011) Antagonistic competition moderates virulence in Bacillus thuringiensis. Ecology letters 14: 765–772. 10.1111/j.1461-0248.2011.01638.x 21635671

[44] Lecadet M-M, Blondel M-O, Ribier J (1980) Generalized transduction in Bacillus thuringiensis var. berliner 1715 using bacteriophage CP-54Ber. Journal of general microbiology 121: 203–212. 7252480 10.1099/00221287-121-1-203

[45] Raymond B, Johnston PR, Wright DJ, Ellis RJ, Crickmore N, et al (2009) A mid-gut microbiota is not required for the pathogenicity of Bacillus thuringiensis to diamondback moth larvae. Environmental microbiology 11: 2556–2563. 10.1111/j.1462-2920.2009.01980.x 19555371

[46] Shelton A, Cooley R, Kroening M, Wilsey W, Eigenbrode S (1991) Comparative analysis of two rearing procedures for diamond-back moth (Lepidoptera: Plutellidae). Journal of entomological science (USA).

[47] Marschner IC (2011) glm2: fitting generalized linear models with convergence problems. The R journal 3: 12–15.

[48] Wickham H (2009) ggplot2: elegant graphics for data analysis: Springer.

[49] Bolker B (2010) bbmle: Tools for general maximum likelihood estimation R package version 0.9.

[50] Cornforth DM, Matthews A, Brown SP, Raymond B (2015) Data from: Bacterial cooperation causes systematic errors in pathogen risk assessment due to the failure of the Independent Action Hypothesis. Dryad Digital Repository. 10.5061/dryad.72f4s PMC4409216 25909384

