# Supplementary material for: Bacterial Cooperation Causes Systematic Errors in Pathogen Risk Assessment due to the Failure of the Independent Action Hypothesis
Source: PLoS Pathog. 2015 Apr 24;11(4):e1004775. doi: 10.1371/journal.ppat.1004775 (PMC4409216; doi:10.1371/journal.ppat.1004775)
Supplement: S1 Text — S1 Table shows comparison of different glm fits to the data in Fig 1A. S2 Table shows the best fit parameters for the best model (based on AIC) among models in S1 Table. (DOCX) [file ppat.1004775.s001.docx]

S1 Table: Eight models fit to the data in Fig. 1A in order to determine which has the lowest AIC for model selection. The first model was selected based on AIC.

| **Model** | **AIC** |
| --- | --- |
| Toxins+log(Spores+1)+Toxins*log(Spores+1) | 143.2 |
| Toxins+log(Spores+1) | 148.6 |
| Toxin+Spores+Toxins*Spores | 191.9 |
| Toxins+Spores | 189.9 |
| log(Spores+1) | 171.8 |
| Spores | 211.6 |
| Toxins | 211.1 |
| 1 | 232.7 |

S2 Table: Parameters of the best logistic model fit to data in Fig. 1A (y~Toxins+log(Spores+1)+Toxins*log(Spores+1)).

| **Parameters** | ***Β*** | **S.E.** | **p-value** |
| --- | --- | --- | --- |
| Intercept | -1.99 | 0.37 | 8.34e-08 |
| Toxins | 0.013 | 0.0028 | 4.90e-06 |
| Log(Spores+1) | 0.37 | 0.063 | 6.62e-09 |
| Toxins*Log(Spores+1) | -0.0013 | 0.00047 | 6.75e-03 |
