# Supplementary figures and images for: Bacterial Cooperation Causes Systematic Errors in Pathogen Risk Assessment due to the Failure of the Independent Action Hypothesis
Source: PLoS Pathog. 2015 Apr 24;11(4):e1004775. doi: 10.1371/journal.ppat.1004775 (PMC4409216; doi:10.1371/journal.ppat.1004775)

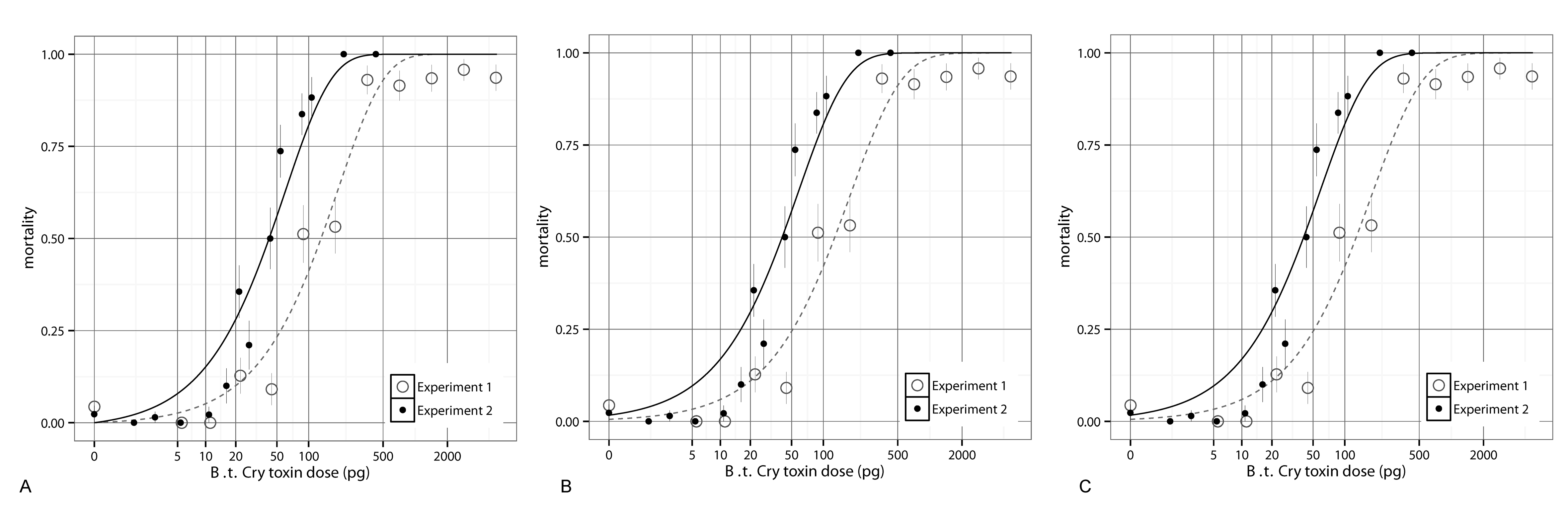

Supplement: S1 Fig — The mortality rates for 900 Bacillus thuringiensis spores supplemented with varying quantities of toxins (+/- S.E.). A) The fit curve from a nonlinear regression with a binomial model (P(k) = 1- (1—p 0)k), using data from both experiments (p 0 = 0.00529 in experiment 1 and p 0 = 0.0163 in experiment 2). The binomial dose-response model assumes no host variability, and unlike the exponential model, doses are assumed exact rather than Poisson distributed. B) The beta-Poisson approximation shown in Eq 2 (α = 8.65, β = 1549.97 in experiment 1 and α = 21.36, β = 1245.22 in experiment 2). C) The exact, confluent hypergeometric form also from Eq 2 α = 8.69, β = 1549.20 in experiment 1 and α = 21.39, β = 1244.07 for experiment 2). In the latter two models, the two parameters are highly correlated when fitting this data, yielding various parameter pairs with nearly identical curves; the fits above are consistent with MCMC runs using log-normal priors. (TIFF) [file ppat.1004775.s002.tiff]
